# Association between Contrast-Enhanced Computed Tomography Radiomic Features, Genomic Alterations and Prognosis in Advanced Lung Adenocarcinoma Patients

**DOI:** 10.3390/cancers15184553

**Published:** 2023-09-14

**Authors:** Lisa Rinaldi, Elena Guerini Rocco, Gianluca Spitaleri, Sara Raimondi, Ilaria Attili, Alberto Ranghiero, Giulio Cammarata, Marta Minotti, Giuliana Lo Presti, Francesca De Piano, Federica Bellerba, Gianluigi Funicelli, Stefania Volpe, Serena Mora, Cristiana Fodor, Cristiano Rampinelli, Massimo Barberis, Filippo De Marinis, Barbara Alicja Jereczek-Fossa, Roberto Orecchia, Stefania Rizzo, Francesca Botta

**Affiliations:** 1 Radiation Research Unit, IEO European Institute of Oncology IRCCS, Via Ripamonti 435, 20141 Milan, Italy; lisa.rinaldi01@universitadipavia.it; 2 Division of Pathology, European Institute of Oncology IRCCS, 20141 Milan, Italy; elena.guerinirocco@ieo.it (E.G.R.); alberto.ranghiero@ieo.it (A.R.); massimo.barberis@ieo.it (M.B.); 3 Department of Oncology and Hemato-Oncology, University of Milan, Via Festa del Perdono 7, 20122 Milan, Italy; stefania.volpe@ieo.it (S.V.);; 4 Division of Thoracic Oncology, IEO European Institute of Oncology IRCCS, Via Ripamonti 435, 20141 Milan, Italy; gianluca.spitaleri@ieo.it (G.S.); ilaria.attili@ieo.it (I.A.); filippo.demarinis@ieo.it (F.D.M.); 5 Department of Experimental Oncology, IEO European Institute of Oncology IRCCS, Via Ripamonti 435, 20141 Milan, Italyfederica.bellerba@ieo.it (F.B.); 6 Division of Radiology, IEO European Institute of Oncology IRCCS, Via Ripamonti 435, 20141 Milan, Italy; marta.minotti87@gmail.com (M.M.); cristiano.rampinelli@ieo.it (C.R.); roberto.orecchia@ieo.it (R.O.); 7 Department of Radiation Oncology, IEO European Institute of Oncology IRCCS, Via Ripamonti 435, 20141 Milan, Italy; 8 Data Management Unit, IEO European Institute of Oncology IRCCS, Via Ripamonti 435, 20141 Milan, Italy; serena.mora@ieo.it (S.M.); cristiana.fodor@ieo.it (C.F.); 9 Scientific Direction, IEO European Institute of Oncology IRCCS, Via Ripamonti 435, 20141 Milan, Italy; 10 Clinica di Radiologia EOC, Istituto Imaging della Svizzera Italiana (IIMSI), Via Tesserete 46, 6900 Lugano, Switzerland; stefaniamariarita.rizzo@eoc.ch; 11 Faculty of Biomedical Sciences, Università della Svizzera italiana, Via G. Buffi 13, 6900 Lugano, Switzerland; 12 Medical Physics Unit, IEO European Institute of Oncology IRCCS, Via Ripamonti 435, 20141 Milan, Italy; francesca.botta@asst-settelaghi.it

**Keywords:** radiomics, lung cancer, CT, genetic alteration, target therapy

## Abstract

**Simple Summary:**

The introduction of targeted therapy has completely changed the treatment options for patients with advanced non-small cell lung cancer (NSCLC). Non-invasive methods to assess mutational status, as well as novel prognostic biomarkers for lung cancer, are thus warranted to improve the management of advanced NSCLC, including adenocarcinoma, and move toward personalized therapy. Radiomics aims to extract high-dimensional features from clinical images in order to find any association with specific clinical endpoints. The aim of this study is to investigate the role of CT radiomics for non-invasive prediction of prognosis and identification of actionable genomic alterations in advanced lung adenocarcinoma patients. Findings from this study support a possible role of CT radiomics in the clinical management of patients with advanced lung cancer; moreover, its findings can contribute to design robust validation studies.

**Abstract:**

Non-invasive methods to assess mutational status, as well as novel prognostic biomarkers, are warranted to foster therapy personalization of patients with advanced non-small cell lung cancer (NSCLC). This study investigated the association of contrast-enhanced Computed Tomography (CT) radiomic features of lung adenocarcinoma lesions, alone or integrated with clinical parameters, with tumor mutational status (*EGFR*, *KRAS*, *ALK* alterations) and Overall Survival (OS). In total, 261 retrospective and 48 prospective patients were enrolled. A Radiomic Score (RS) was created with LASSO-Logistic regression models to predict mutational status. Radiomic, clinical and clinical-radiomic models were trained on retrospective data and tested (Area Under the Curve, AUC) on prospective data. OS prediction models were trained and tested on retrospective data with internal cross-validation (C-index). RS significantly predicted each alteration at training (radiomic and clinical-radiomic AUC 0.95–0.98); validation performance was good for *EGFR* (AUC 0.86), moderate for *KRAS* and *ALK* (AUC 0.61–0.65). RS was also associated with OS at univariate and multivariable analysis, in the latter with stage and type of treatment. The validation C-index was 0.63, 0.79, and 0.80 for clinical, radiomic, and clinical-radiomic models. The study supports the potential role of CT radiomics for non-invasive identification of gene alterations and prognosis prediction in patients with advanced lung adenocarcinoma, to be confirmed with independent studies.

## 1. Introduction

Non-small cell lung cancer (NSCLC) is the leading cause of cancer-related death in the world [1,2]. The advent of targeted therapy (TT) has revolutionized the treatment landscape and prognosis in selected patients with NSCLC who harbor specific driver gene alterations [3,4]. The identification of molecularly specific groups has therefore become essential to adequately tailor treatments and define prognostic categories in patients with advanced NSCLC, including adenocarcinoma. However, different outcomes are observed within the same molecularly defined subgroups, suggesting patterns of disease heterogeneity that need to be more deeply investigated.

Radiomics, based on the extraction of quantitative parameters (i.e., radiomic features) from the radiological images acquired during the clinical practice [5,6,7], is investigated for the ability to characterize different diseases [8,9,10]. In NSCLC, radiomic approaches have been considered for the distinction between benign and malignant lung lesions [11,12] and for associations of radiomic features with treatment response and prognosis [13,14,15,16,17,18,19] or tumor histological [20,21,22] and molecular characteristics [23,24,25,26,27,28,29,30,31,32,33,34]. Indeed, radiomics may offer the opportunity to provide a comprehensive characterization of lung cancer, accounting for the whole tumor heterogeneity in a non-invasive manner [35]. However, to date, radiomics has not yet been translated into clinical applications, due to the still weak reproducibility [36] and lack of independent validation of radiomic models [35,37].

In this study, we investigated the association of radiomic features with genomic alterations and prognosis in a retrospective population of patients affected by lung adenocarcinoma. The radiomic information was extracted from contrast-enhanced CT images, by delineating the whole primary tumor inside the lung. We aimed to develop radiomic and clinical-radiomic models to predict: (i) actionable tumor molecular alteration, focusing on *EGFR* and *KRAS* mutations, and *ALK* gene rearrangement and (ii) patients’ overall survival (OS), exploring whether radiomics can add useful information for physicians’ decisions before and during the treatment of patients with advanced lung adenocarcinoma.

## 2. Materials and Methods

### 2.1. Patient Population

Patients undergoing molecular testing at the Division of Pathology of the European Institute of Oncology IEO (Milan, Italy) between January 2015 and December 2018, with histologically/cytologically confirmed lung adenocarcinoma, were retrospectively retrieved (Dataset 1). Inclusion criteria were: (1) availability of a diagnostic contrast-enhanced CT image; (2) CT images acquired before starting chemotherapy and within ±2 months from biopsy; and (3) no treatment after biopsy and/or CT acquisition. Exclusion criteria were: (1) impossibility of drawing the lung lesion on CT images (e.g., lesion not distinguishable from atelectasis); (2) inadequate CT image or image quality (e.g., unavailability of portal phase images, metal artifacts); (3) coexistence of multiple driver alterations; and (4) unavailability of biopsy date. A second group of patients (Dataset 2) was prospectively enrolled between June 2019 and May 2021, with the same inclusion and exclusion criteria as Dataset 1. The study was conducted in accordance with the Declaration of Helsinki and approved by the Institutional Ethical Committee of the European Institute of Oncology (protocol code R784/18-IEO836). All patients signed informed consent forms for study participation.

For all patients, clinical-pathologic information was retrieved, including tumor volume, age, biological sex, smoking habit, initial lesion site and side, stage, and driver gene alteration status. For Dataset 1, treatments—if any—performed before biopsy and/or CT examination (chemotherapy, radiation therapy, and/or surgery), therapy received after CT examination and follow-up status (alive or deceased) on 31 December 2020 were also collected.

The study was conducted in accordance with the 1964 Helsinki Declaration and subsequent amendments and was approved by the Institutional Review Board and Ethics Committee of the European Institute of Oncology (R784/18-IEO 836). All patients signed informed consent forms for study participation.

### 2.2. Molecular Testing and Radiomic Data

The most frequent actionable gene alterations in NSCLC, including *EGFR* mutations, *KRAS* mutations and *ALK* rearrangements, were assessed.

For Dataset 1 patients, molecular analysis was performed as previously reported [38]. Details are briefly described in the Supplementary Materials, Molecular testing—Dataset 1 paragraph. Similarly, the procedure for molecular analysis adopted for Dataset 2 is summarized in the Supplementary Materials, Molecular testing—Dataset 2 paragraph.

CT images acquired in the portal phase after contrast-medium injection were retrieved. Acquisition and reconstruction data were collected. Three radiologists manually segmented the Volume of Interest (VOI) of the primary lung lesion for each patient with AWServer v.3.2 software (General Electric Healthcare, Waukesha, WI, USA), upon consensus on segmentation criteria. Radiomic features (*n* = 1413) were extracted from each VOI with Pyradiomics v.2.2.0 package [39]. Further details on image parameters, VOI segmentation, and features calculation are reported in the Supplementary Materials, Image analysis paragraph.

### 2.3. Statistical Analysis

#### 2.3.1. Radiomic Features Reproducibility

A preliminary analysis was performed on Dataset 1 to select robust features with respect to CT acquisition and reconstruction settings. After removing 155 features significantly affected by acquisition/reconstruction parameters when extracted from CT images acquired at our Institute [36,40], an analysis of variance (ANOVA) was performed on the remaining 1258 features to identify those exhibiting significantly different distributions when extracted from images acquired at our Institute (*Internal CT*), at different Institution with the same scanner vendor/models as ours (*External CT-same vendor*), and with different scanner vendors/models (*External CT-other vendors*), all present in Dataset 1. Based on the ANOVA results, feature selection and/or patient selection were applied.

#### 2.3.2. Models for Actionable Gene Status Prediction

Three models (radiomic, clinical, and clinical-radiomic) were developed separately for the prediction of the status of each actionable gene (*EGFR* mutations, *KRAS* mutations, *ALK* rearrangements). For each model, Dataset 1 was used as the training set, whereas Dataset 2 served as the validation set. The similarity between the clinical characteristics of the two populations (Dataset 1 and 2) was assessed with the Chi-square or Fisher test for categorical variables, and Student’s *t*-test or Wilcoxon two-independent samples test for continuous variables, as appropriate.

For radiomic model development, a hierarchical cluster analysis was preliminarily applied to exclude redundant features. Then, the Least Absolute Shrinkage and Selection Operator (LASSO)-Logistic regression model was applied to identify the radiomic features mostly associated with the status of each actionable gene. Such features were then combined, the weight of each feature being provided by the model, to create the so-called radiomic score (RS), a score then tested for its ability to predict gene status. For clinical model development, the association between clinical characteristics (age, biological sex, smoking habit, stage, lesion site and side, and treatments before enrollment) and gene status was assessed at univariate analysis. Significant variables were included in a multivariable unconditional logistic regression model and odds ratios (OR) with 95% confidence intervals (CI) were calculated.

A clinical-radiomic model was eventually built with multivariable logistic regression including both RS and the clinical variables.

The validation of each model was then performed on Dataset 2, estimating models’ accuracy with Area Under the Curve (AUC) parameter. The 95% CI for AUCs were calculated using bootstrap resampling.

Finally, an exploratory analysis to look at the association between each calculated RS and relevant clinical variables was performed by using the Spearman correlation coefficient for continuous variables (tumor volume, age) and the Kruskal–Wallis test for categorical variables (sex, smoking history, stage, initial lesion site, and side).

#### 2.3.3. Models for Overall Survival Prediction

Models for OS prediction were trained on Dataset 1. Due to the short follow-up of patients, Dataset 2 could not serve as a validation set, hence internal cross-validation was performed on Dataset 1. Overall survival was calculated as the interval between CT examination and death or last follow-up, updated for all living patients on 31 December 2020. For the radiomic model, hierarchical clustering was first applied, followed by the univariate Cox regression model to select the most representative feature for each cluster (lowest *p*-value). These non-redundant features were included in a LASSO Cox regression model to create the radiomic score (RS). Dataset 1 patients were grouped into high-risk and low-risk groups, using the median RS as the threshold, and Kaplan–Meier survival analysis was performed. The two groups were compared with a log rank test. For the clinical model, Cox proportional hazard regression analysis was applied to identify the clinical variable (age, biological sex, smoking habit, stage, lesion site and side, mutational status, and treatments before and after enrollment) associated with the OS outcome. Both univariate and multivariable analysis were implemented, respectively, to identify variables significantly associated with OS and to build clinical and clinical-radiomic models. The Harrel concordance index (C-index) was calculated to estimate the accuracy of each model after internal 10-fold cross-validation. The analysis was independently repeated 500 times by using different random seeds, and the median C-index with InterQuartile Range (IQR) was reported. Differences between clinical and clinical-radiomic models’ C-index were assessed with the likelihood ratio test.

The whole analysis was also carried out by stratifying patients according to the stage (IV vs. not-IV), and according to the actionable gene status, considering patients with versus without *EGFR* mutation, and patients with versus without *KRAS* mutation.

The statistical analysis was performed using R software, version 4.02.

## 3. Results

### 3.1. Patient and Imaging Characteristics

Among 303 patients of Dataset 1, 285 satisfied the enrollment criteria and were included in the analysis of radiomic feature reproducibility. Imaging parameters are reported in Table S1. Among the 1258 features tested for reproducibility with ANOVA, 145 features were significantly different when extracted from *Internal CT* images versus *External CT-same vendor* images and were excluded. The 24 patients in the *External CT-other vendors* group were also excluded due to the high heterogeneity of acquisition/reconstruction parameters (Table S1). As a result, 1113 radiomic features and 261 patients were included in the final analysis. The flowchart of Dataset 1 patients’ selection is reported in Figure S1.

The clinical-pathological characteristics of Dataset 1 (*n* = 261) and Dataset 2 (*n* = 48) patients are reported in Table 1.

No significant differences were observed between the two populations, except for the stage (*p* = 0.001) and lesion site/side (*p* = 0.04). Among the 261 patients in Dataset 1, 64 had treatments performed before CT examination (39 surgery, 34 radiation therapy, and 56 chemotherapy). Imaging characteristics for Dataset 2 patients were reported in Table S2.

### 3.2. Models for Actionable Gene Status Prediction

Results for the prediction of the *EGFR* mutation are reported in Table S3. The radiomic score was significantly associated with the presence of the *EGFR* mutation at univariate analysis, as well as lesion volume, biological sex, stage, and smoking history. Only lesion volume, sex, and smoking history remained significant in the multivariable clinical model, with less frequent mutation in male patients (OR = 0.27) and in patients with larger lesion volume (OR = 0.99) and more frequent mutation for never- or ex-smokers (OR = 13.80, and OR = 4.00, respectively). Adding the radiomic information to the clinical variables (clinical-radiomic multivariable model), smoking history, and radiomic score were the only significant predictors of *EGFR* mutation (OR = 9.86 for ex-smokers, OR = 15.60 for never smokers, and OR = 3.51 for the radiomic score).

Results for the prediction of the *KRAS* mutation are reported in Table S4. The radiomic score was significantly associated with the presence of the *KRAS* mutation at univariate analysis, as well as age, biological sex, smoking history, and lesion site/side. Only smoking history and lesion site/side remained significant in the multivariable clinical model, with less frequent mutation in never-smoker patients (OR = 0.16) and more frequent mutation in presence of multiple tumor localizations in different lobes (OR = 4.65). Nonetheless, in the clinical-radiomic multivariable model, the radiomic score was the only significant predictor (OR = 35.7).

Results for the prediction of the *ALK* rearrangement are reported in Table S5. The radiomic score was significantly associated with the presence of the *ALK* rearrangement at univariate analysis, as well as age, smoking history, lesion site/side, stage, and previous treatments. In the multivariate clinical model age, smoking history and lesion site/side were confirmed as significant. Patients with the *ALK* gene rearrangement were typically young (OR = 0.90), never-smokers (OR = 21.90), with the lesion in the upper right lobe (OR = 0.21). In the multivariable clinical-radiomic model, age and smoking history were still significant (OR = 0.91 and OR = 37.80, respectively), together with the radiomic score (OR = 3.13).

Previous treatments were not significantly associated with any mutational status in multivariable clinical models.

In the training set, the accuracy was fair to excellent for all the investigated models, with the clinical-radiomic ones exhibiting the highest performance (Table 2).

Independent validation on Dataset 2 confirmed quite good accuracy of clinical and clinical-radiomic models for the prediction of *EGFR* mutations (AUC of 0.85 and 0.86, respectively), whereas AUC remained below 0.65 for *KRAS* mutations and the *ALK* rearrangement (Table 2; Figures S2–S4).

When we assessed the association between each RS and the relevant clinical variables, we found that the RS was correlated with tumor volume for all three genetic mutations, suggesting that all the RS contain some information on tumor size (*p*-values were 0.03 for *EGFR* and <0.0001 for both *KRAS* and *ALK*, results not shown). RS for *KRAS* was also associated with sex (*p* = 0.03, result not shown).

### 3.3. Models for Overall Survival Prediction

Two-hundred forty-nine (249) patients out of 261 were included in the analysis because status on 31 December 2020 was not available for 12 patients. At that date, 79/249 (31.7%) patients were alive, with median (interquartile range) survival after CT examination of 42 (35–52) months. For the deceased patients, the median (range) interval between CT examination and death was 12 (7–23) months.

Treatments delivered to patients after CT examination were chemotherapy (ChT) for 109/249 patients (43.8%), TT for 48/249 patients (19.2%), immune checkpoint inhibitors ICI-based therapy (ICI or ChT plus ICI) for 27/249 patients (10.8%), external beam radiation therapy (EBRT) for 23/249 patients (10.4%), and surgery only for 13/249 patients (5.2%). The information was not available for 29/249 patients (11.6%). In case of the *EGFR* alteration, the first line therapy was gefitinib before October 2019 and osimertininb thereafter; regarding the *ALK* alteration, the first line therapy was crizotinib before June 2018 and alectinib thereafter.

**Figure 1:**
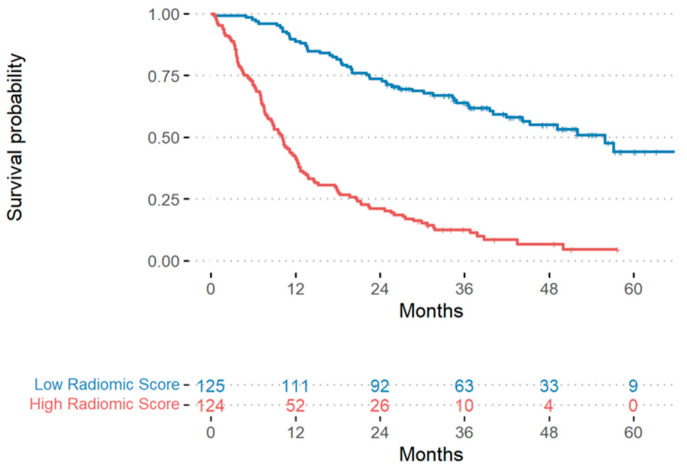
Kaplan–Meier curves for the two groups of patients. The red curve refers to the patients with a high radiomic score (higher than the median value), while the blue curve refers to patients with a low radiomic score (lower than or equal to the median value). For each group of patients, the number of at-risk patients over time (the latter expressed as number of months) is reported below the Kaplan-Maier curves.

For the radiomic model, 63 features were selected by using the LASSO Cox regression model to build the radiomic score. Kaplan–Meier curves for the dichotomized radiomic score are reported in Figure 1, evidencing that a higher radiomic score was significantly associated with lower survival probability (Log-rank test *p*-value < 0.0001).

For the clinical model, stage and type of therapy were confirmed as significantly associated with OS at univariate analysis, with advanced stage (IV) associated with worse prognosis, and TT and ICI-based regimens favoring a better outcome. Both variables were confirmed as independent predictors of OS both in the clinical and clinical radiomic multivariable model. Interestingly, in the latter case, the radiomic score was also an independent predictor of OS (*p* < 0.001).

The accuracy (C-index) of each OS model (radiomic, clinical, and clinical-radiomic) is reported in Table 3. The best performance was obtained with the clinical-radiomic model, suggesting that radiomic features might add further information for the prediction of OS compared to clinical variables alone (LRT test *p* < 0.0001). This result was confirmed after the internal cross-validation (Table 3).

The OS radiomic score obtained from the entire population was a significant independent predictor of OS even when stratifying patients according to stage (Figure S5 for the univariate radiomic model, Table S6 for multivariable radiomic models), and according to the presence/absence of either the *EGFR* or *KRAS* mutation (Table S7 and Table S8, respectively).

## 4. Discussion

In this study, the role of contrast-enhanced CT radiomics in predicting actionable molecular alterations of the tumor and clinical outcome of patients affected by lung adenocarcinoma was investigated.

In our training population, the radiomic score was associated with the presence of selected actionable gene (*EGFR*, *KRAS*, and *ALK*) alterations and OS. Moreover, clinical-radiomic models were developed that outperformed clinical models in the prediction of tumor molecular alterations and OS.

From the clinical perspective, the obtained clinical models confirmed the already established correlations between clinical features, specific gene alterations, and prognosis in NSCLC patients [41,42,43,44], including adenocarcinoma. In addition, our study confirmed that the radiomic score was also an independent predictor of the presence of these gene alterations, as previously investigated in the literature, especially for the *EGFR* mutation [45]. More interestingly, the combined clinical-radiomic score was a stronger predictor for the presence of *EGFR*, *KRAS* and *ALK* gene alterations as compared to models obtained with either clinical or radiomic variables alone. The limited increase of AUC from radiomic to clinical-radiomic models could be partially explained with the fact that the radiomic score seemed to incorporate some relevant clinical information like tumor size or sex (for KRAS), thus RS may be used in the future as a more complex predictor than the clinical information alone.

The preliminary validation on an independent dataset provided promising results especially for the prediction of the *EGFR* mutation (validation AUC 0.78, 0.85, and 0.86 for radiomic, clinical, and clinical-radiomic models). Similar accuracy for *EGFR* mutation prediction was reported in the previous series, with AUC ranging from 0.694 to 0.757 in [28,31,32,34], and AUC up to 0.836 in [31] and 0.85 in [33]. The latter, however, analyzed low-dose CT images [31] and implemented different classification methods than the ones adopted in this study [31,33]. Notably, in these and other studies [32,34], higher accuracy was consistently found when combining radiomic and clinical data, in agreement with our results. Upon validation in larger prospective cohorts, these findings add useful knowledge on the topic and shed light on the potential implications of radiomics in clinical practice. Indeed, despite the central role of tissue testing to obtain diagnosis and molecular data, rejection and failure rates of tissue molecular testing are not negligible, and some patients are unfit to receive or repeat a tumor biopsy [46,47,48,49,50]. In these cases, the use of predictive models combining clinical and radiomic data, in support of other data including those from liquid biopsy [51], might guide tailored testing methods and optimize the testing rate. For the prediction of the *KRAS* mutation and *ALK* arrangement, we obtained validation AUCs equal to 0.64 and 0.59 for radiomic models, 0.62 and 0.60 for clinical models, and 0.61 and 0.65 for clinical-radiomic models. These performances were outperformed by some previously published studies and confirmed by others. Considering the *KRAS* mutation, different studies reported accuracy ranging from 0.71 to 0.94 [31,52,53], in one case [53] obtained with radiomic features from both PET and CT images. Pinheiro et al. [54], instead, did not find any association when extracting features from CT images in a public dataset, in a heterogeneous study in terms of scanning protocol and parameters. Controversial results were also reported for *ALK* rearrangement prediction, with AUC ranging from 0.68 to 0.88 [29,30]. Differences in the patients’ clinical characteristics and the methods for analysis might account for the different reported accuracies. Moreover, the lower validation performance we observed for *KRAS* mutation and *ALK* rearrangement prediction could also be related to the small sample size of the validation population compared to the training population.

In this study, the radiomic score was also found to be an independent predictor for OS, potentially improving the ability of clinical models to separate high-risk and low-risk populations. This finding was consistent during both training and internal validation and also when patients were stratified according to stage or mutational status. In the whole population, we obtained cross-validation C-indexes of 0.79, 0.63, and 0.80 for the radiomic, clinical, and clinical-radiomic models. These results are quite encouraging in comparison to previous studies, reporting sometimes lower validation C-indexes [55,56] that reached 0.75 applying a deep-learning methodology [57]. Moreover, we showed that the radiomic score was an independent predictor of OS also in homogeneous populations according to the presence or absence of specific driver gene alteration, as previously reported [15,17]. Again, despite differences in population and image characteristics, methods for analysis, and types of data associated with radiomic features for models’ creation, previous and current studies suggest that radiomic data might improve the prognostic stratification in lung cancer patients. Nevertheless, these findings need to be further investigated, especially in terms of models’ generalizability.

Among study limitations, we acknowledge that the study population showed some grade of heterogeneity. Despite the proportion of tumors harboring *EGFR*, *ALK,* or *KRAS* alterations being consistent with the frequency reported in lung adenocarcinoma, the reduced subgroup size, especially for ALK-positive cases, might have limited the strength of the models. In addition, our study focused on the *EGFR, ALK* and *KRAS* genes. To date, several other genomic aberrations (i.e., *ROS1*, *BRAF, HER2*, *RET*, *NTRK*) are known as actionable drivers in NSCLC. Although this might result in a heterogeneous non-EGFR/non-ALK/non-KRAS altered population, we analyzed the most frequent actionable and frequently investigated alterations at the time of the study design. Moreover, heterogeneity among treatments (ChT, locoregional only, TT, ICI-based) was also observed, and future larger prospective studies should be designed to focus on selected treatments in more homogeneous populations. Second, the sample size of the validation dataset for mutation prediction was limited. In this regard, the prospective enrollment of the validation cohort is currently ongoing. Furthermore, only internal cross-validation was performed for the OS models, considering the short follow-up of the available prospective cohort. Upon adequate follow-up data of the prospective population becoming available, independent validation will be also performed for the OS models. Third, despite the accurate and robust CT images and features selection, some residual confounding factors may still have affected the radiomic analysis. On the other hand, the restriction to very homogeneous CT images might reduce the generalizability of our models, which will need to be tested on external cohorts of patients including heterogeneous CT images. Fourth, in this study, lesion segmentation was performed manually by three operators. Despite consensus on the segmentation procedure being reached, this might have affected the radiomic feature analysis. In this regard, we are currently investigating the implementation of deep-learning automatic segmentation; a preliminary study from our group demonstrated that the performance of radiomic models for survival prediction in a NSCLC population was comparable considering features extracted from either manual or automatic contours [58]. Last, in this study we did not include the qualitative CT imaging features assessed by visual analysis, which in some studies were found to be associated with mutational status [59,60], since the present study aimed at focusing on the role of radiomics. Future studies might include both feature classes, as well as quantitative parameters extracted from PET images [61], to identify significant features in either group.

## 5. Conclusions

This study provided some evidence of a possible association between CT-based radiomics and lung adenocarcinoma mutational status; in addition, we suggested that CT-based radiomics might improve the prognostic stratification of patients, when adequately integrated with clinical data. Although larger prospective studies are needed to validate and assess the reproducibility of the models, this study adds further indication to support the potential role of radiomic data in the clinical management of patients with advanced lung cancer.

## Figures and Tables

**Table 1:** Clinical-pathologic characteristics of patients included in Dataset 1 and Dataset 2.

	Dataset 1	Dataset 2	
	Mean ± SD	Mean ± SD	*p*-Value ^1^
Tumor volume (cm^3^)	55.4 (± 99.5)	62.4 (±116.0)	0.90
Age (years)	65.9 (±10.4)	65.1 (±12.0)	0.59
	N/Total (%)	N/Total (%)	*p*-value ^1^
Sex			
Female	97/261 (37.2%)	20/48 (41.7%)	0.55
Male	164/261 (62.8%)	28/48 (58.3%)	
Smoking history ^2^			0.55
current smoker	58/252 (23.0%)	9/47 (19.2%)	
ex-smoker	137/252 (54.4%)	24/47 (51.1%)	
never-smoker	57/252 (22.6%)	14/47 (29.8%)	
Initial lesion site and side ^3^			**0.04**
Lower-right	41/256 (16.0%)	8/48 (16.7%)	
Lower-left	30/256 (11.7%)	6/48 (12.5%)	
Medium-right	8/256 (3.1%)	2/48 (4.2%)	
Upper-right	87/256 (33.9%)	18/48 (37.5%)	
Upper-left	80/256 (31.1%)	8/48 (16.7%)	
Mixed	10/256 (3.9%)	6/48 (12.5%)	
Stage			**0.001**
not-IV	102/261 (39.0%)	7/48 (14.6%)	
IV	159/261 (60.9%)	41/48 (85.4%)	
Gene alteration status			
*EGFR* mutation-positive	52/261 (19.9%)	14/48 (29.2%)	0.15
*ALK* rearrangement-positive	22/261 (8.4%)	3/48 (6.25%)	0.78
*KRAS* mutation-positive	106/261 (40.6%)	16/48 (33.3%)	0.34
Others (=no alterations in the three investigated driver genes)	81/261 (31.0%)	15/48 (31.2%)	0.98

^1^ Chi-square or Fisher test, as appropriate, for categorical variables; *t*-test or Wilcoxon two-independent samples test, as appropriate, for continuous variables. Significant *p*-values are in bold. ^2^ Missing data: 9 patients in Dataset 1, 1 patient in Dataset 2; ^3^ Missing data: 5 patients in Dataset 1.

**Table 2:** Gene status prediction. Area Under the Curve (AUC) and 95% confidence intervals (CI) for the prediction of *EGFR*, *ALK* and *KRAS* mutations, during training on Dataset 1 and validation on Dataset 2 are reported. Results are listed separately for the radiomic, clinical and clinical-radiomic models.

Prediction	Model	AUC (95% CI) Training	AUC (95% CI) Validation
EGFR+	Radiomic	0.97 (0.95, 0.99)	0.78 (0.65, 0.91)
	Clinical	0.82 (0.76, 0.87)	0.85 (0.74, 0.95)
	Clinical-Radiomic	0.98 (0.96, 0.99)	0.86 (0.75, 0.96)
KRAS+	Radiomic	0.98 (0.97, 0.99)	0.64 (0.48, 0.80)
	Clinical	0.70 (0.64, 0.76)	0.62 (0.45, 0.79)
	Clinical-Radiomic	0.98 (0.97, 0.99)	0.61 (0.46, 0.77)
ALK+	Radiomic	0.95 (0.92, 0.97)	0.59 (0.29, 0.90)
	Clinical	0.89 (0.80, 0.95)	0.60 (0.23, 0.97)
	Clinical-Radiomic	0.98 (0.96, 0.99)	0.65 (0.31, 1.00)

**Table 3:** Accuracy results of the OS predictive models. The cross-validation results were estimated using the repeated cross-validation technique. The median and the interquartile range among the repeated tests are listed for the cross-validation analysis.

Model	C-Index	C-Index Cross-Validation Median (IQR)
Radiomic	0.78	0.79 (0.71–0.87)
Clinical	0.64	0.63 (0.52–0.72)
Clinical-Radiomic	0.80	0.80 (0.73–0.87)

## Data Availability

Data will be made available upon reasonable request to the corresponding author.

## Supplementary Materials

The following supporting information can be downloaded at: https://www.mdpi.com/article/10.3390/cancers15184553/s1, Table S1: Imaging characteristics of patients included in Dataset 1; Table S2: Imaging characteristics of patients included in Dataset 2; Table S3: Association of radiomic features with the presence of EGFR mutation: univariate and multivariable logistic regression analysis; Table S4: Association of radiomic features with the presence of KRAS mutation: univariate and multivariate logistic regression analysis; Table S5: Association of radiomic features with the presence of ALK rearrangement: univariate and multivariate logistic regression analysis; Table S6: Multivariable Cox regression models predicting Overall Survival with clinical and radiomic features according to stage; Table S7: Multivariable Cox regression models predicting Overall Survival with clinical and radiomic features according to EGFR mutational status; Table S8: Multivariable Cox regression models predicting Overall Survival with clinical and radiomic features according to KRAS mutational status; Figure S1: Flow chart of Dataset 1 patient selection; Figure S2: ROC curve for EGFR mutation prediction according to clinical, radiomic and clinical-radiomic model, and Area Under the Curve (AUC) values obtained during validation on Dataset 2; Figure S3: ROC curve for KRAS mutation prediction according to clinical, radiomic and clinical-radiomic model, and Area Under the Curve (AUC) values obtained during validation on Dataset 2; Figure S4: ROC curve for ALK mutation prediction according to clinical, radiomic and clinical-radiomic model, and Area Under the Curve (AUC) values obtained during validation on Dataset 2; Figure S5: Kaplan–Meier curves for patients separated according to the stage. References [36,40,62,63] are cited in Supplementary Materials.
