# Supplementary material for: Association between Contrast-Enhanced Computed Tomography Radiomic Features, Genomic Alterations and Prognosis in Advanced Lung Adenocarcinoma Patients
Source: Cancers (Basel). 2023 Sep 14;15(18):4553. doi: 10.3390/cancers15184553 (PMC10527057; doi:10.3390/cancers15184553)
Supplement: Supplementary file 1 [file cancers-15-04553-s001.zip › cancers-2578811-supplementary.pdf]

# Supplementary Materials: Association between Contrast-Enhanced CT Radiomic Features, Genomic Alterations and Prognosis in Advanced NSCLC Patients

**Lisa Rinaldi, Elena Guerini Rocco, Gianluca Spitaleri, Sara Raimondi, Ilaria Attili, Alberto Ranghiero, Giulio Cammarata, Marta Minotti, Giuliana Lo Presti, Francesca De Piano, Federica Bellerba, Gianluigi Funicelli, Stefania Volpe, Serena Mora, Cristiana Fodor, Cristiano Rampinelli, Massimo Barberis, Filippo De Marinis, Barbara Alicja Jereczek-Fossa, Roberto Orecchia, Stefania Rizzo and Francesca Botta**

**Molecular testing—Dataset 1**

DNA was extracted from formalin-fixed, paraffin-embedded tumor tissue samples (FFPE) or tumor cytological samples and subjected to next-generation sequencing analysis using the CE-IVD Oncomine Solid Tumour DNA assay (ThermoFisher, Waltham, Massachusetts, USA) for the evaluation of the mutational status (single-nucleotide variants and small insertions and deletions (InDels)) of 22 genes, following the manufacturer's instructions. Briefly, 10 ng of DNA was used for the library and template preparation on the Ion Chef System (ThermoFisher Scientific, Waltham, MA, USA). The sequencing run was performed on the Ion S5 System (ThermoFisher Scientific, Waltham, MA, USA) and data were analyzed with the Ion Reporter Analysis Software (ThermoFisher Scientific, Waltham, MA, USA). The evaluation of ALK and ROS1 gene rearrangements was performed by fluorescence in situ hybridization (FISH) using ALK and ROS1 dual-colour probe (IQFISH Break Apart Probe Agilent Technologies, Santa Clara, California, USA) and evaluated according to the manufacturer criteria. In ambiguous or equivocal cases, ALK or ROS1 immunohistochemical staining (clone D5F3, Ventana, Tucson, Arizona, USA and clone D4D6, respectively) was performed.

**Molecular testing—Dataset 2**

DNA and RNA were automatically extracted from tumor cytology or tissue specimen using Promega Maxwell RSC DNA or RNA FFPE kit (Promega, Madison, WI, USA) and subjected to NGS using a panel targeting relevant hotspots mutations, copy number gains and fusions across 161 cancer-related genes (Oncomine Comprehensive Assay Thermofisher Scientific, Waltham, MA). The sequencing run was performed on the Ion S5 System (ThermoFisher Scientific, Waltham, MA, USA) and data were analyzed with the Ion Reporter Analysis Software (ThermoFisher Scientific, Waltham, MA, USA). Only alterations with a Variant Allele Frequency (VAF) equal/superior to 5% and with adequate quality metrics (read depth > 100; VAF x read depth > 25; p-value = 0.00001 and Median of the Absolute values of all Pairwise Difference (MAPD) < 0.5) were considered for further analyses(1). Fusion genes detected by NGS were further confirmed using FISH and/or immunohistochemistry.

**Image analysis**

For each image, the following acquisition and reconstruction data were collected: scanner vendor, scanner model, tube voltage, axial pixel size, slice thickness, matrix size, and reconstruction information when available (algorithm, kernel, parameters).

The segmentation criteria adopted by the radiologist for VOI drawing were the following: exclusion of vessels, inclusion of opacity along the lesion edges, and use of either mediastinal (width: 350HU, level: 40 HU) or lung (width: 1500 HU, level: -600 HU) windows settings according to lesion position.

The extracted radiomic features (n=1413) belonged to shape, intensity (first-order) and texture (grey level co-occurrence matrix, grey level dependence matrix, grey level run

length matrix, grey level size zone matrix, and neighbouring grey tone difference matrix) categories. Among them, 153 features were computed from unprocessed images (referred to as original), 560 after application of Wavelet transform (choosing the Coif1 as wavelet type), and 700 after application of Laplacian Of Gaussian (LoG) filter with different sigma values (0.5, 1.0, 1.5, 2.5, and 5 mm). Feature extraction and correction due to their dependence on the number of voxels in the VOI were performed as previously described(2,3). According to IBSI recommendations(4), discretization of grey-level intensities (25HU fixed bin width) and voxel resampling (0.78 mm pixel in the axial plane, and 2.5 mm slice thickness whenever acquired differently) were applied before feature extraction.

## References


1. Passaro A, Attili I, Rappa A, Vacirca D, Ranghiero A, Fumagalli C, et al. Genomic Characterization of Concurrent Alterations in Non-Small Cell Lung Cancer (NSCLC) Harboring Actionable Mutations. Cancers. 2021;13:2172.
2. Rinaldi L, De Angelis SP, Raimondi S, Rizzo S, Fanciullo C, Rampinelli C, et al. Reproducibility of radiomic features in CT images of NSCLC patients: an integrative analysis on the impact of acquisition and reconstruction parameters. European Radiology Experimental. 2022;6:2.
3. Rinaldi L, Pezzotta F, Santaniello T, De Marco P, Bianchini L, Origgi D, et al. HeLLePhant: A phantom mimicking non-small cell lung cancer for texture analysis in CT images. Physica Medica. 2022;97:13–24.
4. Zwanenburg A, Vallières M, Abdalah MA, Aerts HJWL, Andrearczyk V, Apte A, et al. The Image Biomarker Standardization Initiative: Standardized Quantitative Radiomics for High-Throughput Image-based Phenotyping. Radiology. 2020;295:328–38.


**Table S1.** Imaging characteristics of patients included in Dataset 1. All images were acquired in helical mode with 512 × 512 matrix size and were reconstructed with a standard kernel.

<table>
<tr><th>Group</th><th>Scanner vendor</th><th>Scanner model</th><th>Tube voltage (kVp)</th><th>Axial pixel size (mm) median (min-max)</th><th>Slice thickness (mm) / Spacing between slices (mm)</th><th>Reconstruction algorithm</th></tr>
<tr><td>Internal CT:<br>224 pts</td><td>GE (224 pts)</td><td>Optima 660 (116 pts)<br>Discovery 750 (99 pts)<br>Lightspeed (9 pts)</td><td>100 kVp (29 pts)<br>120 kVp (183 pts)<br>140 kVp (12 pts)</td><td>0.77 (0.62-0.96)</td><td>2.5/2.5 (224 pts)</td><td>ASIR*40 (76 pts)<br>ASIR50 (41 pts)<br>ASIR60 (80 pts)<br>ASIR70 (18 pts)<br>FBP (9 pts)</td></tr>
<tr><td>External CT-same vendor:<br>37 pts</td><td>GE (37 pts)</td><td>Optima 660 (10 pts)<br>Discovery 750 (2 pts)<br>Lightspeed (14 pts)<br>Brightspeed (7 pts)<br>Revolution (4 pts)</td><td>120 kVp (37 pts)</td><td>0.80 (0.64-0.98)</td><td>2.5/2.5 (36 pts)<br>3.8/3.8 (1 pt)</td><td>ASIR30 (3 pts)<br>ASIR40 (7 pts)<br>ASIR50 (5 pts)<br>ASIR60 (3 pts)<br>FBP (19 pts)</td></tr>
<tr><td rowspan="2">External CT-other vendors:<br>24 pts</td><td>Siemens (18 pts)</td><td>Emotion (2 pts)<br>Sensation (3 pts)<br>Somaton (13 pts)</td><td rowspan="2">100 kVp (3 pts)<br>120 kVp (15 pts)<br>130 kVp (5 pts)<br>140 kVp (1 pt)</td><td rowspan="2">0.75 (0.66 – 0.88)</td><td rowspan="2">2.5/2.5 (4 pts)<br>3/3 (20 pts)</td><td>B20s (1 pt); B30f (2 pts);<br>B31f (6 pts); B31s (6 pts);<br>I30f (1pt); I40f (2 pts)</td></tr>
<tr><td>Philips (6 pts)</td><td>Brilliance (5 pts)<br>Ingenuity (1 pt)</td><td>iDose3 (1 pt);<br>iDose4 (1 pt);<br>iDose5 (1 pt);<br>FBP (3 pts)</td></tr>
</table>

* Adaptive Statistical Iterative Reconstruction algorithm.

**Table S2.** Imaging characteristics of patients included in Dataset 2.

| Group | Scanner vendor | Scanner model | Tube voltage (kVp) | Axial pixel size (mm) median (min-max) | Slice thickness (mm) / Spacing between slices (mm) | Reconstruction algorithm |
|---|---|---|---|---|---|---|
| Internal CT: 44 pts | GE (44 pts) | Optima 660 (20 pts) Discovery 750 (24 pts) | 100 kVp (25 pts) 120 kVp (19 pts) | 0.78 (0.70-0.98) | 2.5/2.5 (44 pts) | ASIR*50 (20 pts) ASIR60 (24 pts) |
| External CT-same vendor: 2 pts | GE (2 pts) | Revolution (2 pts) | 120 kVp (2 pts) | 0.70 (1 pt) 0.82 (1 pt) | 2.5/2.5 (1 pt) 1.25/1.25 (1 pt) | ASIR50 (1 pt) ASIR60 (1 pt) |
| External CT-other vendors: 2 pts | Siemens (2 pts) | Emotion (1 pt) Somaton (1 pt) | 120 kVp (1 pt) 130 kVp (1 pt) | 0.76 (1 pt) 0.80 (1 pt) | 3/3 (2 pts) | B30s (1 pt); I40f (1 pt) |

* Adaptive Statistical Iterative Reconstruction algorithm.

**Table S3.** Association of radiomic features with the presence of EGFR mutation: univariate and multivariable logistic regression analysis.

| | EGFR - N (%) | EGFR + N (%) | Univariate Odds Ratio (95%CI)* | Clinical Multivariable Odds Ratio + (95%CI) | Clinical-Radiomic Multivariable Odds Ratio + (95%CI) |
|---|---|---|---|---|---|
| **Lesion volume (cm$^3$)^** | 61.6 (±106.8) | 23.3 (±27.8) | **0.99 (0.98, 1.00)** | **0.99 (0.98, 1.00)** | 1.00 (0.99, 1.02) |
| **Age (years)^** | 65.8 (±10.2) | 66.1 (±11.4) | 1.00 (0.97, 1.03) | | |
| **Sex** | | | | | |
| Female | 65/209 (31.1%) | 32/52 (61.5%) | 1.00 | 1.00 | 1.00 |
| Male | 144/209 (68.9%) | 20/52 (38.5%) | **0.28 (0.15, 0.53)** | **0.27 (0.13, 0.55)** | 0.32 (0.09, 1.08) |
| **Smoking history** | | | | | |
| Current | 55/203 (27.1%) | 3/49 (6.1%) | 1.00 | 1.00 | 1.00 |
| Ex-smoker | 117/203 (57.6%) | 20/49 (40.8%) | 3.13 (0.89, 11.10) | **4.00 (1.09, 14.70)** | **9.86 (1.28, 76.30)** |
| No | 31/203 (15.3%) | 26/49 (53.1%) | **15.38 (4.30, 54.90)** | **13.80 (3.66, 52.0)** | **15.60 (2.03, 120)** |
| **Initial lesion site and side^** | | | | | |
| Lower-right | 33/205 (16.1%) | 8/51 (15.7%) | 1.00 | | |
| Lower-left | 27/205 (13.2%) | 3/51 (5.9%) | 0.46 (0.11, 1.90) | | |
| Medium-right | 6/205 (2.9%) | 2/51 (3.9%) | 1.38 (0.23, 8.13) | | |
| Upper-right | 64/205 (31.2%) | 23/51 (45.1%) | 1.48 (0.60, 3.67) | | |
| Upper-left | 66/205 (32.2%) | 14/51 (27.5%) | 0.87 (0.33, 2.29) | | |
| Mixed | 9/205 (4.4%) | 1/51 (2.0 %) | 0.46 (0.05, 4.16) | | |
| **Stage** | | | | | |
| Not IV | 90/209 (42.6%) | 12/52 (25.0%) | 1.00 | 1.00 | 1.00 |
| IV | 119/209 (56.9%) | 40/52 (76.9%) | **2.27 (1.14, 4.50)** | 1.90 (0.87, 4.12) | 2.57 (0.68, 9.68) |
| **Previous treatments[1]** | | | | | |
| No | 162/209 (77.5%) | 35/52 (67.3%) | 1.00 | | |
| Yes | 47/209 (22.5%) | 17/52 (32.7%) | 1.67 (0.86, 3.25) | | |
| **Previous surgery** | | | | | |
| No | 181/209 (86.6%) | 41/52 (78.8%) | 1.00 | | |
| Yes | 28/209 (13.4%) | 11/52 (21.2%) | 1.73 (0.80, 3.77) | | |
| **Previous radiotherapy** | | | | | |
| No | 182/209 (87.1%) | 45/52 (86.5%) | 1.00 | | |
| Yes | 27/209 (12.9%) | 7/52 (13.5%) | 1.05 (0.43, 2.45) | | |
| **Previous chemotherapy** | | | | | |
| No | 167/209 (79.9%) | 38/52 (73.1%) | 1.00 | | |
| Yes | 42/209 (20.1%) | 14/52 (26.9%) | 1.46 (0.73, 2.95) | | |
| **Radiomic Score** | -415.77 (±2.60) | -410.08 (±2.38) | **3.52 (2.47, 5.02)** | - | **3.51 (2.36, 5.55)** |

CI= Confidence Interval; Note: significant ORs and p-values are in bold; ^Mean (± SD); * Univariate logistic regression; + Multivariate logistic regression; [1]Tratments before CT examination; ^ Missing data about site and side: 4 patients in EGFR- group, 1 patient in EGFR+ group.

**Table S4.** Association of radiomic features with the presence of KRAS mutation: univariate and multivariate logistic regression analysis.

| | KRAS - N (%) | KRAS + N (%) | Univariate Odds Ratio (95%CI)* | Clinical Multivariate Odds Ratio+ (95%CI) | Clinical- Radiomic Multivariate Odds Ratio+ (95%CI) |
|---|---|---|---|---|---|
| **Lesion volume ($cm^3$)^** | 42.2 (±73.5) | 71.1 (±123.0) | 1.00 (1.00, 1.01) | 1.00 (1.00, 1.01) | 1.00 (1.00, 1.00) |
| **Age (years)^** | 64.9 (±11.3) | 67.3 (±8.9) | **1.02 (1.00, 1.05)** | 1.01 (0.99, 1.04) | 1.04 (0.98, 1.10) |
| **Sex** | | | | | |
| Female | 66/155 (42.6%) | 31/106 (29.2%) | 1.00 | 1.00 | 1.00 |
| Male | 89/155 (57.4%) | 75/106 (70.8%) | **1.79 (1.07, 3.06)** | 1.37 (0.77, 2.47) | 0.72 (0.19, 2.63) |
| **Smoking history** | | | | | |
| Current | 28/150 (18.7%) | 30/102 (29.4%) | 1.00 | 1.00 | 1.00 |
| Ex-smoker | 72/150 (48.0%) | 65/102 (63.7%) | 0.84 (0.45, 1.56) | 0.89 (0.46, 1.72) | 0.66 (0.16, 2.52) |
| No | 50/150 (33.3%) | 7/102 (6.9%) | **0.13 (0.05, 0.32)** | **0.16 (0.06, 0.40)** | 0.27 (0.02, 2.31) |
| **Initial lesion site and side** | | | | 1.00 (not Mixed) | 1.00 (not Mixed) |
| Lower-right | 27/152 (17.8%) | 14/104 (13.5%) | 1.00 | | |
| Lower-left | 15/152 (9.9%) | 15/104 (14.4%) | 1.93 (0.74, 5.13) | | |
| Medium-right | 6/152 (3.9%) | 2/104 (1.9%) | 0.64 (0.09, 3.23) | | |
| Upper-right | 56/152 (36.8%) | 31/104 (29.8%) | 1.07 (0.49, 2.37) | | |
| Upper-left | 46/152 (30.3%) | 34/104 (32.7%) | 1.43 (0.66, 3.17) | | |
| Mixed | 2/152 (1.3%) | 8/104 (7.7%) | **7.71 (1.67, 55.93)** | **4.65 (1.01, 34.20)** | 11.50 (0.53, 764.0) |
| **Stage** | | | | | |
| Not IV | 58/155 (37.4%) | 44/106 (41.5%) | 1.00 | | |
| IV | 97/155 (62.6%) | 62/106 (58.5%) | 0.87 (0.52, 1.44) | | |
| **Previous treatments[1]** | | | | | |
| No | 113/155 (72.9%) | 84/106 (79.2%) | 1.00 | | |
| Yes | 42/155 (27.1%) | 22/106 (20.8%) | 0.70 (0.39, 1.26) | | |
| **Previous surgery** | | | | | |
| No | 131/155 (84.5%) | 91/106 (85.8%) | 1.00 | | |
| Yes | 24/155 (15.5%) | 15/106 (14.2%) | 0.90 (0.44, 1.79) | | |
| **Previous radiotherapy** | | | | | |
| No | 134/155 (86.5%) | 93/106 (87.7%) | 1.00 | | |
| Yes | 21/155 (13.5%) | 13/106 (12.3%) | 0.89 (0.42, 1.85) | | |
| **Previous chemotherapy** | | | | | |
| No | 119/155 (76.8%) | 86/106 (81.1%) | 1.00 | | |
| Yes | 36/155 (23.2%) | 20/106 (18.9%) | 0.77 (0.41, 1.41) | | |
| **Radiomic Score** | -0.77 (±1.03) | 2.09 (±1.18) | **30.90 (13.10,94.60)** | - | **35.7 (14.0, 124.0)** |

CI= Confidence Interval; Note: significant ORs and p-values are in bold; ^Mean (± SD); * Univariate logistic regression; + Multivariate logistic regression; [1]Tratments before CT examination.

**Table S5.** Association of radiomic features with the presence of ALK rearrangement: univariate and multivariate logistic regression analysis.

| | ALK - N (%) | ALK + N (%) | Univariate Odds Ratio (95%CI)* | Clinical Multivariate Odds Ratio+ (95%CI) | Clinical-Radiomic Multivariate Odds Ratio+ (95%CI) |
|---|---|---|---|---|---|
| **Lesion volume (cm³)^** | 52.84 (±96.9) | 65.95 (±105.4) | 1.00 (1.00, 1.01) | 1.00 (1.00, 1.01) | 1.00 (1.00, 1.01) |
| **Age (years)^** | 66.84 (±9.6) | 55.46 (±13.1) | **0.91 (0.88, 0.95)** | **0.90 (0.85, 0.95)** | **0.91 (0.84, 0.98)** |
| **Sex** | | | | | |
| Female | 85/239 (35.6%) | 12/22 (54.5%) | 1.00 | | |
| Male | 154/239 (64.4%) | 10/22 (45.5%) | 0.46 (0.19, 1.11) | | |
| **Smoking history** | | | | | |
| Current | 57/231 (24.7%) | 1/21 (4.8%) | 1.00 | 1.00 | 1.00 |
| Ex-smoker | 130/231 (56.3%) | 7/21 (33.3%) | **3.07 (0.53, 58.06)** | 6.34 (0.64, 63.25) | 8.86 (0.61, 367) |
| No | 44/231 (19.0%) | 13/21 (61.9%) | **16.84 (3.17, 311.74)** | **21.90 (2.27, 212.00)** | **37.80 (2.73, 1702)** |
| **Initial lesion site and side** | | | | 1.00 (not upper-right) | 1.00 (not upper-right) |
| Lower-right | 35/235 (14.9%) | 6/21 (28.6%) | 1.00 | | |
| Lower-left | 26/235 (11.1%) | 4/21 (19.0%) | 0.90 (0.21, 3.47) | | |
| Medium-right | 7/235 (3.0%) | 1/21 (4.8%) | 0.83 (0.04, 6.05) | | |
| Upper-right | 84/235 (35.7%) | 3/21 (14.3%) | **0.21 (0.04, 0.84)** | **0.21 (0.05, 0.96)** | 0.19 (0.08, 74.2) |
| Upper-left | 73/235 (31.1%) | 7/21 (33.33%) | 0.56 (0.17, 1.85) | | |
| Mixed | 10/235 (4.3%) | 0/21 (0.00%) | Not calculable | | |
| **Stage** | | | | | |
| Not IV | 99/239 (41.4%) | 3/22 (13.6%) | 1.00 | | |
| IV | 140/239 (58.6%) | 19/22 (86.4%) | **4.56 (1.50, 19.76)** | | |
| **Previous treatments[1]** | | | | | |
| No | 176/239 (73.6%) | 21/22 (95.5%) | 1.00 | | |
| Yes | 63/239 (26.4%) | 1/22 (4.5%) | **0.13 (0.01, 0.66)** | | |
| **Previous surgery** | | | | | |
| No | 202/239 (84.5%) | 20/22 (90.9%) | 1.00 | | |
| Yes | 37/239 (15.5%) | 2/22 (9.1%) | 0.55 (0.08, 1.98) | | |
| **Previous radiotherapy** | | | | | |
| No | 207/239 (86.6%) | 20/22 (90.9%) | 1.00 | | |
| Yes | 32/239 (13.4%) | 2/22 (9.1%) | 0.65 (0.10, 2.36) | | |
| **Previous chemotherapy** | | | | | |
| No | 186/239 (77.8%) | 19/22 (86.4%) | 1.00 | | |
| Yes | 53/239 (22.2%) | 3/22 (13.6%) | 0.55 (0.13, 1.70) | | |
| **Radiomic Score** | 21.08 (±2.39) | 25.62 (±1.38) | **2.76 (1.99, 4.26)** | - | **3.13 (2.00, 5.97)** |

CI= Confidence Interval; Note: significant ORs and p-values are in bold; ^Mean (± SD); * Univariate logistic regression; + Multivariate logistic regression; [1]Tratments before CT examination

**Table S6.** Multivariable Cox regression models predicting Overall Survival with clinical and radiomic features according to stage.

| | Stage IV | | | | Stage Not-IV | | | |
|---|---|---|---|---|---|---|---|---|
| | Clinical Multivariable | | Clinical-Radiomic Multivariable | | Clinical Multivariable | | Clinical-Radiomic Multivariable | |
| | Hazard Ratio (95% CI) | p-value | Hazard Ratio (95% CI) | p-value | Hazard Ratio (95% CI) | p-value | Hazard Ratio (95% CI) | p-value |
| **Treatment** | | | | | | | | |
| ChT | 0.58 (0.14-2.39) | 0.448 | 0.48 (0.12-2.00) | 0.316 | 0.49 (0.23-1.04) | 0.064 | 0.66 (0.31-1.41) | 0.283 |
| TT | 0.34 (0.08-1.44) | 0.143 | 0.26 (0.06-1.09) | 0.066 | 0.26 (0.07-0.99) | **0.048** | 0.51 (0.14-1.91) | 0.319 |
| ICI-based | 0.48 (0.10-2.19) | 0.34 | 0.43 (0.09-2.01) | 0.286 | 0.29 (0.10-0.89) | **0.03** | 0.37 (0.12-1.14) | 0.083 |
| EBRT | 0.54 (0.12-2.48) | 0.43 | 0.65 (0.14-2.99) | 0.578 | 0.24 (0.06-0.88) | **0.031** | 0.40 (0.11-1.51) | 0.177 |
| Surgery only | -- | -- | -- | -- | -- | -- | -- | -- |
| Not available | 0.80 (0.19-3.46) | 0.77 | 0.50 (0.12-2.20) | 0.362 | 0.59 (0.16-2.21) | 0.438 | 1.05 (0.28-3.94) | 0.942 |
| **Radiomic Score** | | | 3.77 (2.87-4.96) | **<0.001** | | | 5.12 (3.28-7.99) | **<0.001** |

**Table S7.** Multivariable Cox regression models predicting Overall Survival with clinical and radiomic features according to EGFR mutational status.

| | EGFR + | | | | EGFR - | | | |
|---|---|---|---|---|---|---|---|---|
| | Clinical Multivariable | | Clinical-Radiomic Multivariable | | Clinical Multivariable | | Clinical-Radiomic Multivariable | |
| | Hazard Ratio (95% CI) | p-value | Hazard Ratio (95% CI) | p-value | Hazard Ratio (95% CI) | p-value | Hazard Ratio (95% CI) | p-value |
| **Stage at diagnosis** | | | | | | | | |
| IV | -- | -- | -- | -- | -- | -- | -- | -- |
| Not-IV | 0.45 (0.16-1.28) | 0.135 | 0.84 (0.29-2.42) | 0.75 | 0.38 (0.26-0.56) | **<0.001** | 0.43 (0.29-0.64) | **<0.001** |
| **Treatment** | | | | | | | | |
| ChT | 0.10 (0.01-0.64) | **0.016** | 0.42 (0.06-2.92) | 0.38 | 0.54 (0.26-1.10) | 0.088 | 0.62 (0.30-1.29) | 0.20 |
| TT | 0.05 (0.01-0.31) | **0.002** | 0.11 (0.02-0.70) | **0.02** | 0.28 (0.11-0.73) | **0.009** | 0.27 (0.10-0.71) | **0.008** |
| ICI-based | 0.37 (0.03-5.19) | 0.46 | 0.09 (0.01-1.43) | 0.09 | 0.40 (0.17-0.94) | **0.036** | 0.59 (0.25-1.39) | 0.23 |
| EBRT | nc | nc | nc | nc | 0.34 (0.13-0.84) | **0.02** | 0.56 (0.22-1.41) | 0.21 |
| Surgery only | -- | -- | -- | -- | -- | -- | -- | -- |
| Not available | 0.03 (0.00-0.30) | **0.003** | 0.06 (0.01-0.54) | **0.013** | 1.01 (0.45-2.28) | 0.98 | 0.89 (0.38-2.07) | 0.79 |
| **Radiomic Score** | | | 4.61 (2.31-9.21) | **<0.001** | | | 4.17 (3.22-5.41) | **<0.001** |

nc=not calculable (no EGFR+ patient treated with EBRT).

**Table S8.** Multivariable Cox regression models predicting Overall Survival with clinical and radiomic features according to KRAS mutational status.

| | KRAS + | | | | KRAS - | | | |
|---|---|---|---|---|---|---|---|---|
| | **Clinical Multivariable** | | **Clinical-Radiomic Multivariable** | | **Clinical Multivariable** | | **Clinical-Radiomic Multivariable** | |
| | **Hazard Ratio (95% CI)** | **p-value** | **Hazard Ratio (95% CI)** | **p-value** | **Hazard Ratio (95% CI)** | **p-value** | **Hazard Ratio (95% CI)** | **p-value** |
| **Stage at diagnosis** | | | | | | | | |
| IV | -- | -- | -- | -- | -- | -- | -- | -- |
| Not-IV | 0.35 (0.21-0.61) | **<0.001** | 0.35 (0.19-0.63) | **<0.001** | 0.43 (0.26-0.71) | **0.001** | 0.52 (0.31-0.86) | **0.011** |
| **Treatment** | | | | | | | | |
| ChT | 0.40 (0.18-0.87) | **0.021** | 0.46 (0.20-1.05) | 0.065 | 0.69 (0.20-2.32) | 0.55 | 0.75 (0.22-2.53) | 0.65 |
| TT | | | | | 0.35 (0.10-1.24) | 0.104 | 0.36 (0.10-1.32) | 0.123 |
| ICI-based | 0.39 (0.15-1.06) | 0.065 | 0.47 (0.17-1.30) | 0.15 | 0.38 (0.09-1.56) | 0.178 | 0.45 (0.11-1.85) | 0.27 |
| EBRT | 0.50 (0.14-1.73) | 0.27 | 1.50 (0.44-5.13) | 0.52 | 0.39 (0.10-1.49) | 0.17 | 0.51 (0.13-1.98) | 0.33 |
| Surgery only | -- | -- | -- | -- | -- | -- | -- | -- |
| Not available | 0.62 (0.23-1.72) | 0.36 | 0.42 (0.14-1.24) | 0.11 | 0.80 (0.21-3.02) | 0.75 | 0.79 (0.21-3.00) | 0.73 |
| **Radiomic Score** | | | 4.97 (3.40-7.46) | **<0.001** | | | 3.59 (2.67-4.83) | **<0.001** |

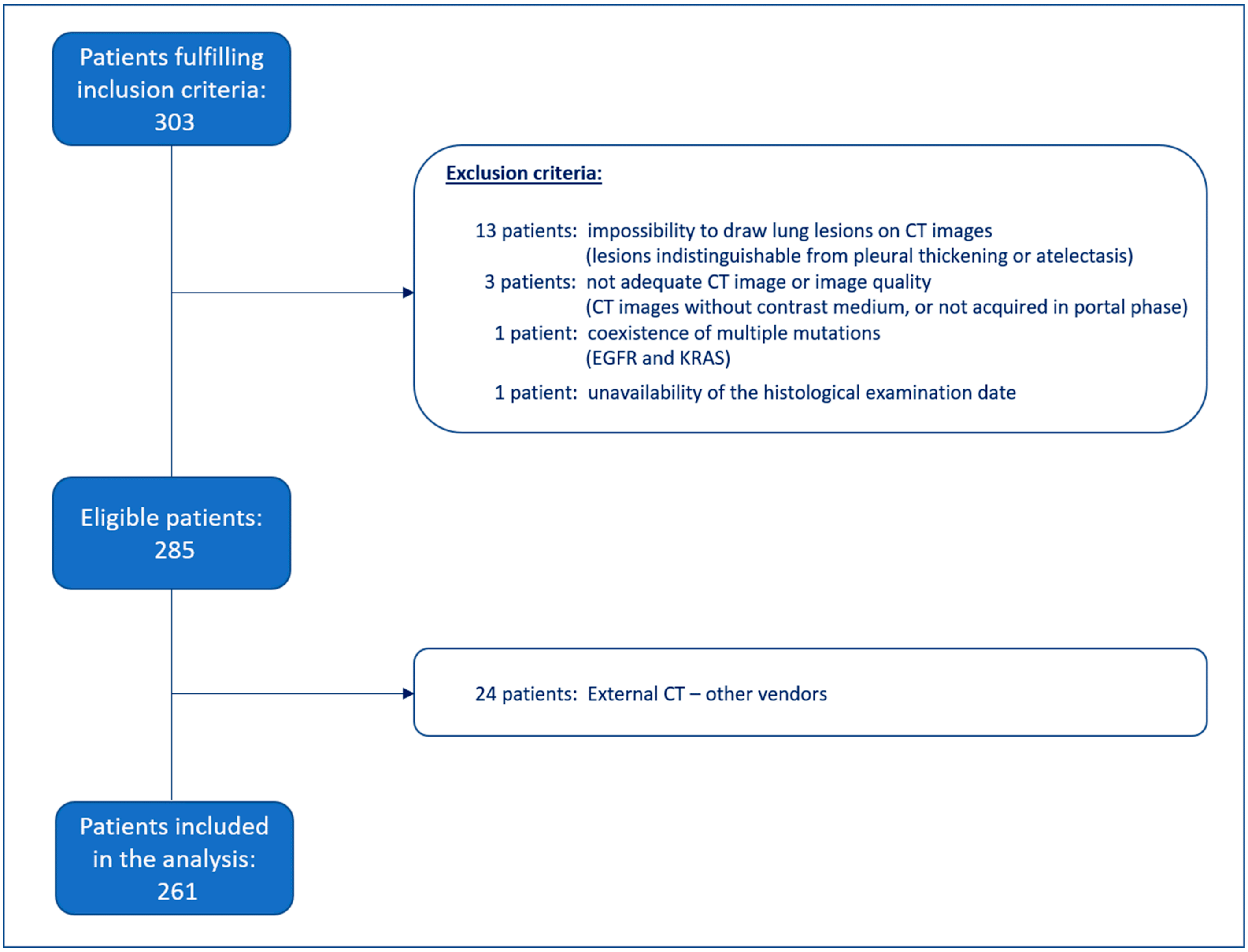


**Figure S1.** Flow chart of Dataset 1 patient selection.

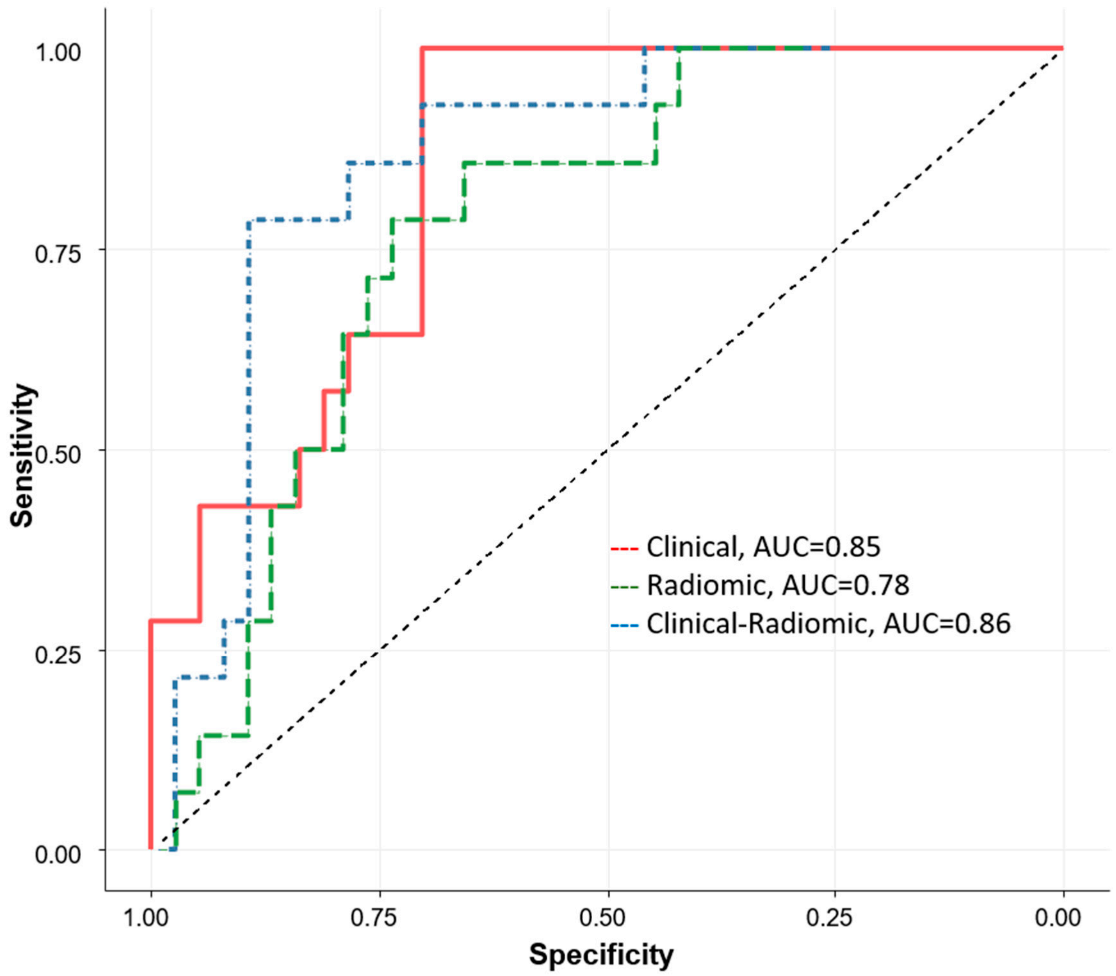


**Figure S2.** ROC curve for EGFR mutation prediction according to clinical, radiomic and clinical-radiomic model, and Area Under the Curve (AUC) values obtained during validation on Dataset 2.

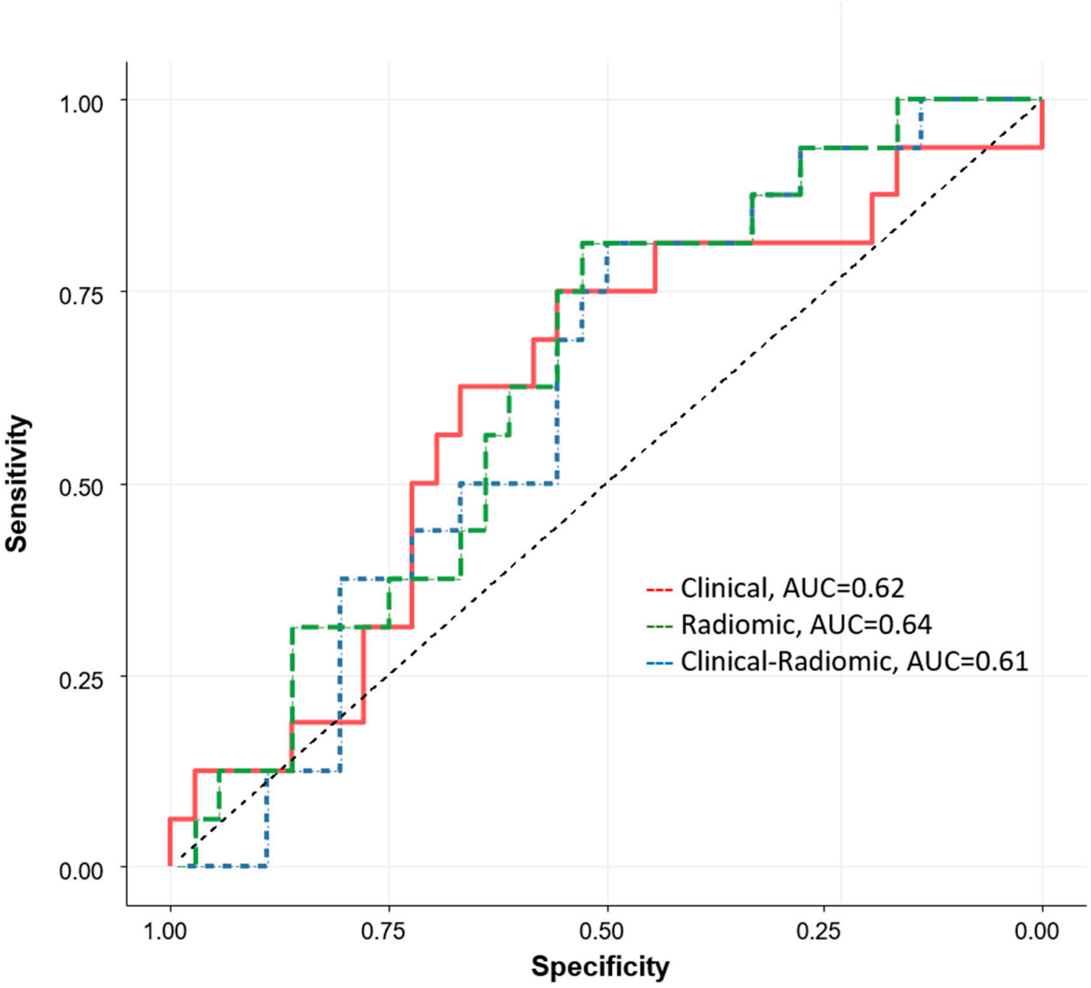


**Figure S3.** ROC curve for KRAS mutation prediction according to clinical, radiomic and clinical-radiomic model, and Area Under the Curve (AUC) values obtained during validation on Dataset 2.

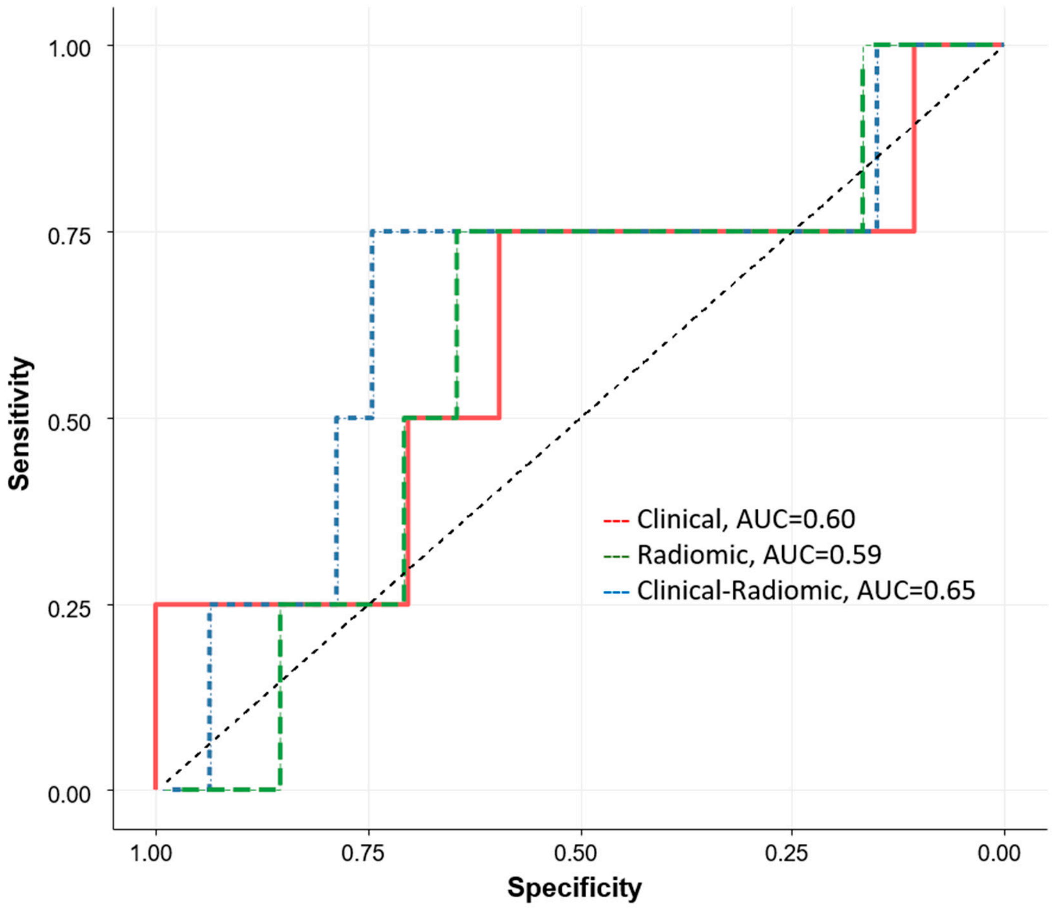


**Figure S4.** ROC curve for ALK mutation prediction according to clinical, radiomic and clinical-radiomic model, and Area Under the Curve (AUC) values obtained during validation on Dataset 2.

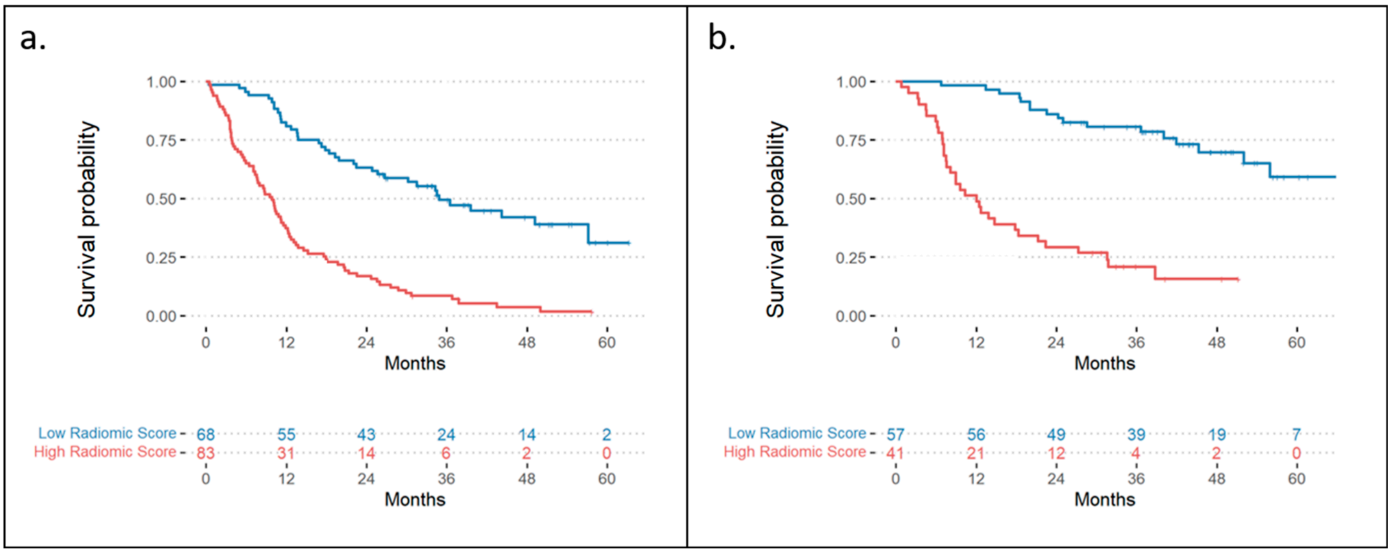


**Figure S5.** Kaplan–Meier curves for patients separated according to the stage: **a.** stage IV and **b.** stage not-IV. For each population, the red curve refers to the patients with a high radiomic score (higher than the median value), while the blue curve refers to patients with a low radiomic score (lower than or equal to the median value). In both cases, the separation between high-risk and low-risk populations was statistically significant ($p<0.0001$).
